# Forebrain excitatory neuron-specific SENP2 knockout mouse displays hyperactivity, impaired learning and memory, and anxiolytic-like behavior

**DOI:** 10.1186/s13041-020-00591-8

**Published:** 2020-04-14

**Authors:** Dehua Huang, Huiqing Liu, Aoxue Zhu, Yi Zhou, Yong Li

**Affiliations:** 1 Department of Biochemistry and Molecular Cell Biology, Shanghai Key Laboratory for Tumor Microenvironment and Inflammation, Shanghai Jiao Tong University School of Medicine, 280 South Chongqing Road, Shanghai, 200025 China; 2 Department of Biomedical Sciences, College of Medicine, Florida State University, Tallahassee, FL USA

**Keywords:** SENP2, Conditional knockout mice, Anxiety-like behavior, Learning and memory, RNA-seq

## Abstract

Sentrin/SUMO-specific protease 2 (SENP2) is a member of SENPs family involved in maturation of SUMO precursors and deSUMOylation of specific target, and is highly expressed in the central nervous system (CNS). Although SENP2 has been shown to modulate embryonic development, fatty acid metabolism, atherosclerosis and epilepsy, the function of SENP2 in the CNS remains poorly understood. To address the role of SENP2 in the CNS and its potential involvement in neuropathology, we generated SENP2 conditional knockout mice by crossing floxed SENP2 mice with CaMKIIα-Cre transgenic mice. Behavioral tests revealed that SENP2 ablation induced hyper-locomotor activity, anxiolytic-like behaviors, spatial working memory impairment and fear-associated learning defect. In line with these observations, our RNA sequencing (RNA-seq) data identified a variety of differential expression genes that are particularly enriched in locomotion, learning and memory related biologic process. Taken together, our results indicated that SENP2 plays a critical role in emotional and cognitive regulation. This SENP2 conditional knockout mice model may help reveal novel mechanisms that underlie a variety of neuropsychiatric disorders associated with anxiety and cognition.

## Introduction

SUMOylation is a dynamic and reversible post-translational modification that modulates diverse functions of target proteins, including protein stability, protein subcellular localization, protein-protein or protein-DNA interactions, and protein kinase activity [1]. Cellular abundance of particular SUMO-conjugated substrates is regulated by a balance between SUMO conjugation and SUMO deconjugation. Sentrin-specific proteases (SENPs) catalyze the removal of SUMO from SUMO-conjugated target proteins as well as the cleavage of SUMO from its precursor proteins, thus playing a critical role in regulating the SUMOylation level of targets [2, 3]. In mammals, the SENP family consists of six members, which can be divide into three groups (SENP1 and SENP2; SENP3 and SENP5; SENP6 and SENP7) based on homology and function analysis [3]. We recently showed that SENP1 participates in regulating nociceptive signaling in models of inflammatory pain and attenuates I/R injury induced cell death in a transient brain ischemia/reperfusion mouse model [4, 5]. Moreover, SENP2 has been reported to play a role in cardiac development [6], neuronal survive [7] and seizure [8]. According to the Allen Brain Atlas, SENP2 mRNAs are highly expressed in the forebrain [9], but the function of SENP2 in the central nervous system (CNS) remains unclear. As SENP2 is required for expression of key developmental genes, global deletion of SENP2 is embryonically lethal [6–8, 10]. Thus, we developed a forebrain excitatory neuron-specific SENP2 knockout mouse model to examine SENP2 functions in the CNS. We found that these conditional knockout (cKO) animals display hyperactivity and reduced anxiety-like behavior, impaired learning and memory. Gene ontology (GO) analysis of the differential expression genes revealed enrichment for numerous cellular and molecular functional categories, including those related to “Cell death” and “Immune response”. Taken together, our results indicate that SENP2 plays an important role in the forebrain, and its absence leads to molecular and behavioral changes associated with locomotion, anxiety, learning and memory.

## Results

### Generation of forebrain-specific SENP2 cKO mice

**Fig. 1:**
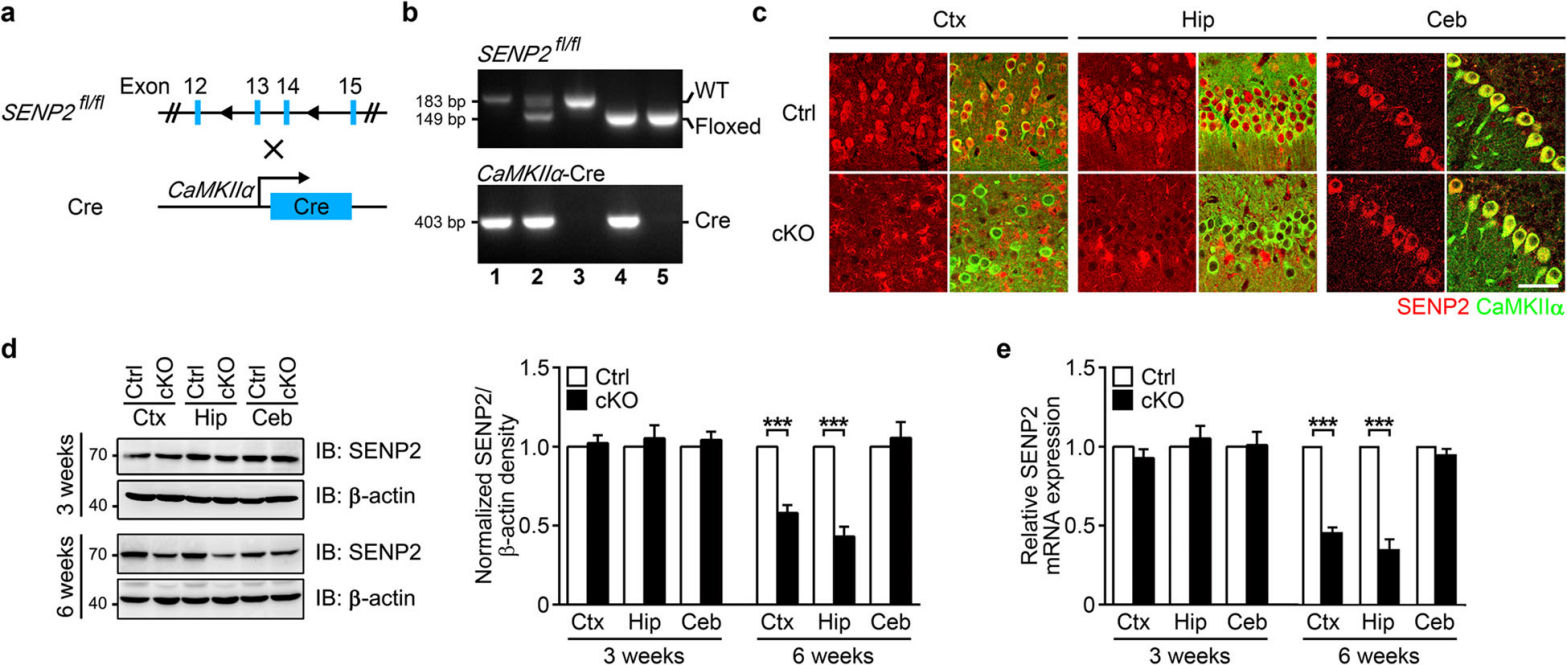
Generation of forebrain-specific SENP2 cKO mice. **a** The schematic diagram of SENP2 cKO mouse generation. **b** Genotyping identification of conditional knockouts by PCR. **c** Confocal microscopy photomicrographs showing double immunostaining of CaMKIIα (green) and SENP2 (red) in excitatory neurons of 6-week-old cKO and littermate control mice. There is significantly less SENP2 positive excitatory neurons in cortex (Ctx) and hippocampus (Hip), but not in cerebellum (Ceb) brain slices. Scale bar = 50 μm. **d** In 6-week-old cKO mice, As detected by western blots, the SENP2 protein level is significantly reduced in the Ctx and Hip, but not in the Ceb of 6-week-old SENP2 cKO mice (left panel). Quantification of the Western blots is shown in the right panel. **e** As detected by qPCR, the SENP2 mRNA level is significantly reduced in Ctx and Hip, but not in the Ceb of 6-week-old cKO mice. All data are presented as mean ± SEM. Ctrl (Control): *n* = 3; cKO (SENP2 conditional knockout): *n* = 3. Statistical analysis performed with two-way ANOVA followed by Bonferroni’s post-hoc. *p < 0.05, **p < 0.01, ***p < 0.001 compared with littermate controls

To determine the functional role of SENP2 in the CNS, we crossed floxed SENP2 (*SENP2*^*fl/fl*^) mice [8] with CaMKIIα-Cre transgenic mice [11] to generate SENP2 conditional knockout (cKO) mice, in which SENP2 was selectively removed from principal neurons of postnatal forebrain (Fig. 1a-b). Because the CaMKIIα-Cre transgene is expressed between postnatal days 14–21 in excitatory neurons in the forebrain [11], this allowed us to specifically assess SENP2 function in the postnatal forebrain without disrupting its contribution to early CNS development and/or causing embryonic lethality. In the cKO mice, SENP2 protein levels were strongly reduced in the forebrain (cortical and hippocampal) excitatory neurons (Fig. 1c). As determined by western blot analyses, loss of SENP2 protein expression occurred in the cortex (58.01 ± 4.90% of littermate controls) and hippocampus (42.96 ± 6.24% of littermate controls) of 6-week-old cKO mice, but not in the cerebellum (105.66 ± 9.99% of littermate controls), where the Cre recombinase is not expressed (Fig. 1d). Additionally, real-time quantitative PCR (qPCR) determined that SENP2 mRNA levels in the cKO mice were significantly reduced in cortex (45.82 ± 2.94% of littermate controls) and hippocampus (35.17 ± 6.07% of littermate controls) of 6-week-old cKO mice. However, no change was observed in the cKO mice at 3 weeks after birth (Fig. 1e).

### Behavioral analyses of cKO mice

**Fig. 2:**
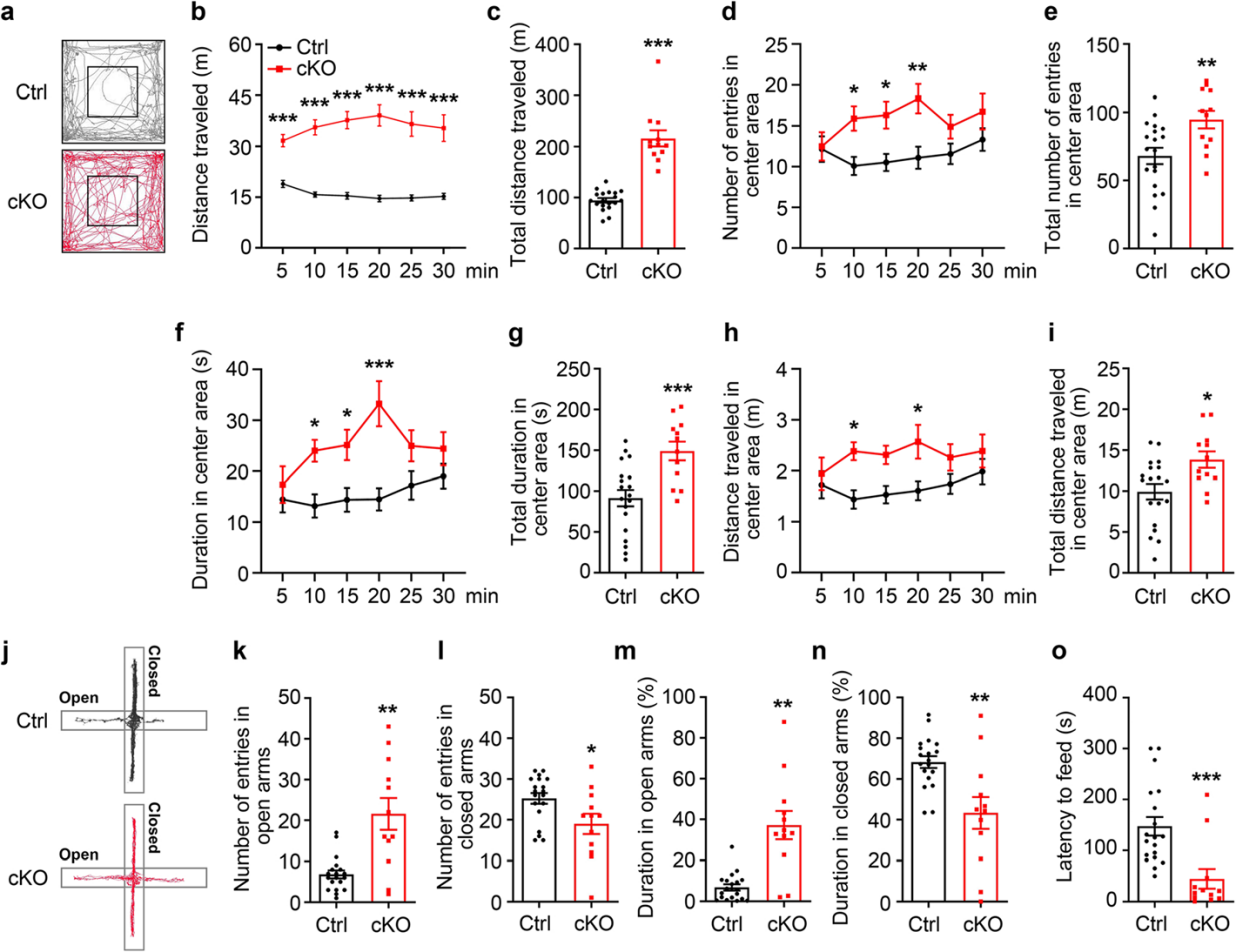
SENP2 cKO mice exhibited hyperactivity and decreased anxiety-like behavior. **a** Representative exploratory tracks of either littermate control (Ctrl) or cKO mice in the open field. **b**-**i** Analysis of open field exploration behavior for **b** distance traveled (m): increased locomotor activity of cKO mice at every 5 min block (5 min: Ctrl: 18.91 ± 1.03, *n* = 19; cKO: 31.69 ± 1.81, *n* = 12; *p* < 0.0001; 10 min: Ctrl: 15.76 ± 0.73, *n* = 19; cKO: 35.64 ± 2.22, *n* = 12; *p* < 0.0001; 15 min: Ctrl: 15.40 ± 0.96, *n* = 19; cKO: 37.76 ± 2.53, *n* = 12; *p* < 0.0001; 20 min: Ctrl: 14.60 ± 0.96, *n* = 19; cKO: 39.16 ± 3.13, *n* = 12; *p* < 0.0001; 25 min: Ctrl: 14.75 ± 0.88, *n* = 19; cKO: 36.61 ± 3.62, *n* = 12; *p* < 0.0001; 30 min: Ctrl: 15.24 ± 0.88, *n* = 19; cKO: 35.40 ± 3.92, *n* = 12; *p* < 0.0001; two-way ANOVA analysis followed by Bonferroni’s post-hoc), and **c** increased locomotor activity of cKO mice in the 30-min open field test (Ctrl: 94.66 ± 4.43, *n* = 19; cKO: 216.26 ± 15.81, *n* = 12; *p* < 0.0001, Welch’s *t*-test). **d** Analysis of number of entries in center area at every 5 min: Increased number of entries of cKO mice in center area at 10, 15 and 20 min (5 min: Ctrl: 12.16 ± 1.56, *n* = 19; cKO: 12.50 ± 1.73, *n* = 12; *p* > 0.9999; 10 min: Ctrl: 10.11 ± 1.11, *n* = 19; cKO: 15.92 ± 1.49, *n* = 12; *p* = 0.0422; 15 min: Ctrl: 10.53 ± 1.05, *n* = 19; cKO: 16.33 ± 1.65, *n* = 12; *p* = 0.0425; 20 min: Ctrl: 11.11 ± 1.35, *n* = 19; cKO: 18.33 ± 1.78, *n* = 12; *p* = 0.0052; 25 min: Ctrl: 11.58 ± 1.27, *n* = 19; cKO: 14.92 ± 1.45, *n* = 12; *p* = 0.7143; 30 min: Ctrl: 13.33 ± 1.35, *n* = 19; cKO: 16.75 ± 2.18, *n* = 12; *p* = 0.6865; two-way ANOVA analysis followed by Bonferroni’s post-hoc), and **e** increased number of entries of cKO mice in the 30-min open field test (Ctrl: 68.11 ± 6.00, *n* = 19; cKO: 94.75 ± 6.48, *n* = 12; *p* = 0.0068, Student’s t-test). **f** Analysis of duration spent (s) in the center of the field at every 5 min: An increased center time was observed in cKO mice at 10, 15 and 20 min (5 min: Ctrl: 14.44 ± 2.52, *n* = 19; cKO: 17.35 ± 3.57, *n* = 12; *p* > 0.9999; 10 min: Ctrl: 13.17 ± 2.30, *n* = 19; cKO: 24.00 ± 12.14, *n* = 12; *p* = 0.0477; 15 min: Ctrl: 14.37 ± 2.34, *n* = 19; cKO: 25.12 ± 3.00, *n* = 12; *p* = 0.0497; 20 min: Ctrl: 14.47 ± 2.19, *n* = 19; cKO: 33.24 ± 4.43, *n* = 12; *p* < 0.0001; 25 min: Ctrl: 17.20 ± 2.77, *n* = 19; cKO: 25.00 ± 3.07, *n* = 12; *p* = 0.3334; 30 min: Ctrl: 19.00 ± 2.37, *n* = 19; cKO: 24.40 ± 3.26, *n* = 12; *p* > 0.9999; two-way ANOVA analysis followed by Bonferroni’s post-hoc), and **g** increased time spent (s) of cKO mice in the center of the field in the 30-min open field test (Ctrl: 91.64 ± 9.97, *n* = 19; cKO: 149.04 ± 11.42, *n* = 12; *p* = 0.0009, Student’s *t*-test). **h** Analysis of distance traveled (m) in center area at every 5 min: Increased distance traveled of cKO mice in the center area at 10 and 20 min (5 min: Ctrl: 1.72 ± 0.26, *n* = 19; cKO: 1.94 ± 0.32, *n* = 12; *p* > 0.9999; 10 min: Ctrl: 1.44 ± 0.18, *n* = 19; cKO: 2.38 ± 0.18, *n* = 12; *p* = 0.0379; 15 min: Ctrl: 1.53 ± 0.17, *n* = 19; cKO: 2.31 ± 0.18, *n* = 12; *p* = 0.1428; 20 min: Ctrl: 1.61 ± 0.19, *n* = 19; cKO: 2.57 ± 0.33, *n* = 12; *p* = 0.0324; 25 min: Ctrl: 1.74 ± 0.19, *n* = 19; cKO: 2.26 ± 0.26, *n* = 12; *p* = 0.7768; 30 min: Ctrl: 1.88 ± 0.26, *n* = 19; cKO: 2.39 ± 0.32, *n* = 12; *p* > 0.9999; two-way ANOVA analysis followed by Bonferroni’s post-hoc), and **i** Increased distance traveled (m) in center area of the field in the 30-min open field test (Ctrl: 9.93 ± 0.95 m, *n* = 19; cKO: 13.85 ± 1.00 m, *n* = 12; *p* < 0.0107, Student’s *t*-test). **j** Representative track of exploration in elevated plus maze of either littermate control (Ctrl) or cKO mice. **k, i** Analysis of number of entries in open arms or closed arms: more number of cKO mice entries in open arms (Ctrl: 6.84 ± 0.97, *n* = 19; cKO: 21.58 ± 3.91, *n* = 12; *p* = 0.0031, Welch’s *t*-test), **l** less number of cKO mice entries in closed arms (Ctrl: 25.26 ± 1.32, *n* = 19; cKO: 19.00 ± 2.49, *n* = 12; *p* = 0.0213, Student’s *t*-test), **m**, **n** Analysis of percentage of time spent in open arms or closed arms: more percentage of time spent in closed arms of cKO mice (Ctrl: 6.85 ± 1.54%, *n* = 19; cKO: 37.20 ± 6.90%, *n* = 12; *p* = 0.0010, Welch’s *t*-test), **n** less percentage of time spent in closed arms of cKO mice (Ctrl: 68.31 ± 2.94%, *n* = 19; cKO: 43.36 ± 7.81%, *n* = 12; *p* = 0.0096, Welch’s *t*-test). **o** Novelty suppressed feeding. cKO mice have less latency (s) to feeding compared with littermate control mice (Ctrl: 294.79 ± 36.24, *n* = 19; cKO: 89.42 ± 38.73, *n* = 12; *p* = 0.0008, Student’s *t*-test). All data presented as mean ± S.E.M.

We performed a battery of behavioral tests to evaluate the behavioral phenotype of SENP2 cKO mice. In open field test, cKO mice travelled more distance (m) than their littermate controls during a 30 min recording period (Ctrl (Control): 94.66 ± 4.43, *n* = 19; cKO: 216.26 ± 15.81, *n* = 12; *p* < 0.0001, Welch’s *t*-test) (Fig. 2a-c). As abnormal exploratory behaviors may also be indicative of changes in anxiety, we assessed the number of entries, duration spent and distance traveled in center area. We observed that SENP2 cKO mice entered the center more frequently (Ctrl: 68.11 ± 6.00, *n* = 19; cKO: 94.75 ± 6.48, *n* = 12; *p* = 0.0068, Student’s *t*-test) (Fig. 2d-e), spent more time (s) in center area (91.64 ± 9.97, *n* = 19; cKO: 149.04 ± 11.42, *n* = 12; *p* = 0.0009, Student’s *t*-test) (Fig. 2f-g) and traveled more distance in center area than littermate controls (Ctrl: 9.93 ± 0.95, *n* = 19; cKO: 13.85 ± 1.00, *n* = 12; *p* < 0.0107, Student’s *t*-test) in a 30 min test session (Fig. 2h-i). Although a 5 min test session is often sufficient to assess the critical components of general exploratory locomotion, the most commonly used measure of overall exploratory/locomotor activity is the total distance traveled. SENP2 cKO mice spent longer time and entered more frequency in the center area than littermate control mice at 10 min, 15 min, 20 min(Fig. 2d, f), and traveled more distance at 10 min, 20 min (Fig. 2h), although no statistically significant difference between cKO and littermate control mice was observed at 5 min, 25 min and 30 min. These findings suggest that cKO mice showed great aspiration for exploring the center area, and thus displayed decreased anxiety-like behavior.

To further explore the anxiety-like behaviors, cKO mice were evaluated in two anxiety-related behavioral assays including elevated plus maze and novelty suppressed feeding. In the evaluated plus maze test, cKO mice gained more number of entries in the open arms (Ctrl: 6.84 ± 0.97, *n* = 19; cKO: 21.58 ± 3.91, *n* = 12; *p* = 0.0031, Welch’s *t*-test) (Fig. 2k) and less number of entries in the closed arms accordingly (Ctrl: 25.26 ± 1.32, *n* = 19; cKO: 19.00 ± 2.49, *n* = 12; *p* = 0.0213, Student’s *t*-test) (Fig. 2l). In addition, cKO mice spent more percentage of time in the open arms (Ctrl: 6.85 ± 1.54%, *n* = 19; cKO: 37.20 ± 6.90%, *n* = 12; *p* = 0.0010, Welch’s *t*-test) (Fig. 2m) and less percentage of time in the closed arms (Ctrl: 68.31 ± 2.94%, *n* = 19; cKO: 43.36 ± 7.81%, *n* = 12; *p* = 0.0096, Welch’s *t*-test) (Fig. 2n). Moreover, cKO mice significantly reduced the latency (s) to feed in the novel environment compared with littermate controls (Ctrl: 294.79 ± 36.24, *n* = 19; cKO: 89.42 ± 38.73, *n* = 12; *p* = 0.0008, Student’s *t*-test) (Fig. 2o). Taken together, these results demonstrated that cKO mice exhibited anxiolytic-like behavior.

**Fig. 3:**
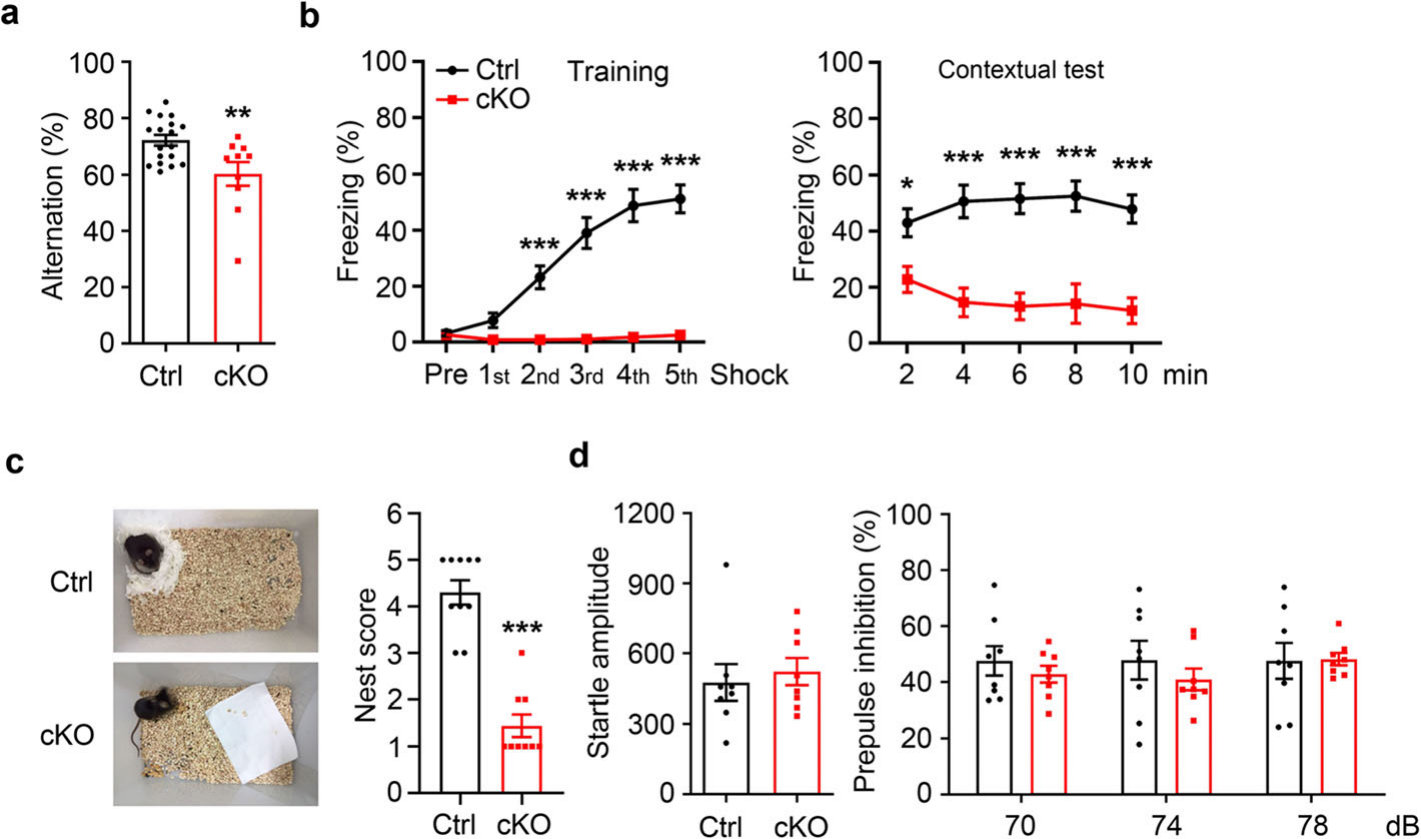
SENP2 ablation impaired working memory, contextual fear learning and nest building activity. **a**-**b** Cognitive test of cKO mice. **a** Y maze test. cKO mice showed reduced percentage of accurate spontaneous alternations over total number of alternations among the three arms (Ctrl: 72.18 ± 1.84%, *n* = 18; cKO: 60.35 ± 4.22%, *n* = 10; *p* = 0.0062, Student’s *t*-test). **b** Contextual fear condition test. cKO mice showed low levels of freezing behaviors in training session (pre-shock: Ctrl: 3.25 ± 0.94%, *n* = 19; cKO: 2.66 ± 1.47%, *n* = 11, *p* > 0.9999; 1st shock: Ctrl: 7.83 ± 2.59%, *n* = 19; cKO: 0.89 ± 0.52%, *n* = 11, *p* > 0.9999; 2nd shock: Ctrl: 23.20 ± 4.06%, *n* = 19; cKO: 0.86 ± 0.48%, *n* = 11, *p* = 0.0009; 3rd shock: Ctrl: 39.05 ± 5.48%, *n* = 19; cKO: 1.11 ± 0.63%, *n* = 11, *p* < 0.0001; 4th shock: Ctrl: 48.83 ± 5.80%, *n* = 19; cKO:1.87 ± 1.11%, *n* = 11, *p* < 0.0001; 5th shock: Ctrl: 51.21 ± 4.96%, *n* = 19; cKO: 2.61 ± 1.01%, *n* = 11, *p* < 0.0001; two-way ANOVA analysis followed by Bonferroni’s post-hoc). In contextual fear retrieval session, cKO mice display very lowly freezing behaviors (2 min: Ctrl: 42.92 ± 4.97%, *n* = 19; cKO: 21.79 ± 4.21%, *n* = 11, *p* = 0.0481; 4 min: Ctrl: 50.61 ± 5.81%, *n* = 19; cKO: 14.55 ± 5.14%, *n* = 11, *p* < 0.0001; 6 min: Ctrl: 51.57 ± 5.35%, *n* = 19; cKO: 13.10 ± 4.79%, *n* = 11, *p* < 0.0001; 8 min: Ctrl: 52.49 ± 5.41%, *n* = 19; cKO: 14.05 ± 7.04%, *n* = 11, *p* < 0.0001; 10 min: Ctrl: 47.86 ± 5.02%, *n* = 19; cKO: 11.57 ± 4.60%, *n* = 11, *p* < 0.0001; two-way ANOVA analysis followed by Bonferroni’s post-hoc). **c** Nest building test. cKO mice display lower nest scores in the nest building test (Ctrl: 4.30 ± 0.26, *n* = 10; cKO: 1.44 ± 0.24, *n* = 9; *p* < 0.0001, Student’s *t*-test). **d** Acoustic startle response and prepulse inhibition in cKO mice. cKO mice display normal startle reflex in 120 dB acoustic stimulus compared with littermate controls (Ctrl: 475.71 ± 78.32, *n* = 8; cKO: 522.48 ± 58.08, *n* = 8; *p* = 0.6389, Student’s t-test) and showed similar PPI of the startle response than littermate control mice at the prepulse level of 70 dB, 74 dB, 78 dB (70 dB: Ctrl: 47.64 ± 5.23%, *n* = 8; cKO: 42.90 ± 3.01%, *n* = 8; *p* > 0.9999; 74 dB: Ctrl: Ctrl: 47.86 ± 6.92%, *n* = 8; cKO: 41.00 ± 3.84%, *n* = 8; *p* = 0.9895; 78 dB: Ctrl: 47.63 ± 6.44%, *n* = 8; cKO: 48.23 ± 2.21%, *n* = 8; *p* > 0.9999; two-way ANOVA analysis followed by Bonferroni’s post-hoc). All data presented as mean ± S.E.M.

Previous studies have suggested that SUMOylation plays an important role in learning and memory [12–14]. Given the critical function of SENP2 in regulating SUMOylation status and its high-level expression in the brain, we investigate whether the loss of SENP2 in forebrain excitatory neurons impairs learning and memory. As first, we assessed spatial working and reference memory of cKO mice using the Y-maze spontaneous alternation task. Compared with littermate controls, cKO mice displayed significantly reduced alternations (Ctrl: 72.18 ± 1.84%, *n* = 18; cKO: 60.35 ± 4.22%, *n* = 10; *p* = 0.0062, Student’s *t*-test) (Fig. 3a), suggesting an impairment of spatial working memory in cKO mice. Moreover, we examined associative learning and memory behaviors using a contextual fear conditioning protocol [15]. As shown in Fig. 3b, littermate control mice responded well in training session and exhibited freezing behavior when reintroduced to the same context 24 h later. In contrast, cKO mice not only showed impaired learning ability during training, but also displayed extremely low freezing level in contextual test session, demonstrating an impairment of cKO mice in associative learning and memory. Furthermore, we found that the cKO mice had a substantially decreased nesting score in the nest building assay compared with littermate control mice (Ctrl: 4.30 ± 0.26, *n* = 10; cKO: 1.44 ± 0.24, *n* = 9; *p* < 0.0001, Student’s *t*-test) (Fig. 3c). In rodents, the nest building behavior represents a form of social behaviors, and that impaired nest building is considered to represent a negative phenotype of psychiatric diseases including schizophrenia [16]. To determine whether cKO mice displayed other core characteristics of the neuropsychiatric disorders, we conducted prepulse inhibition (PPI) task to measure the sensorimotor gating of cKO mice [17]. Compared with their littermates, cKO mice displayed normal startle reaction (Ctrl: 475.71 ± 78.32, *n* = 8; cKO: 522.48 ± 58.08, *n* = 8; *p* = 0.6389, Student’s *t*-test) (Fig. 3d). When assayed in a prepulse inhibition (PPI) task, there were no differences between cKO and littermate control mice in the extent of PPI at 3 increasing prepulse sound intensities (%PPI of 70 dB prepulse stimulus: Ctrl: 47.64 ± 5.23%, *n* = 8; cKO: 42.90 ± 3.01%, *n* = 8; *p* > 0.9999; %PPI of 74 dB prepulse stimulus: Ctrl: 47.86 ± 6.92%, *n* = 8; cKO: 41.00 ± 3.84%, *n* = 8; *p* = 0.9895; %PPI of 78 dB prepulse stimulus: Ctrl: 47.63 ± 6.44%, *n* = 8; cKO: 48.23 ± 2.21%, *n* = 8; *p* > 0.9999; two-way ANOVA analysis followed by Bonferroni’s post-hoc) (Fig. 3d). Thus, cKO mice had no obvious deficit in PPI.

### Identification of SENP2-regulated transcripts in the cerebral cortex

**Fig. 4:**
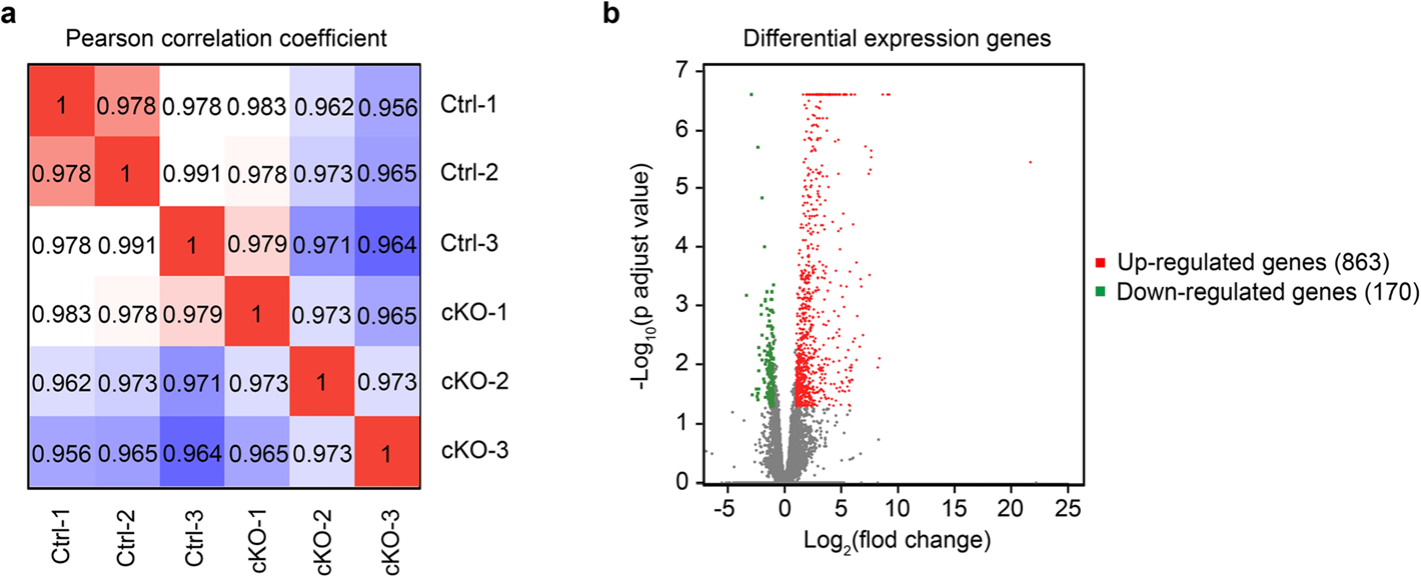
RNA-seq analysis of cKO. **a** The pearson correlation coefficient between cKO and littermate control mice. Results showed that correlation coefficient of 3 pairs cortical samples from cKO and littermate control mice was more than 0.95. **b** The differential expression genes between cKO and littermate control mice. The red dots represent up-regulated 863 genes, while the green dots represent down-regulated 170 genes using the most stringent criteria (intersection of adjusted *p* < 0.05 and the relative gene expression level > 2 fold change, the gene was considered as the differential expression gene)

To elucidate the molecular mechanism underlying the behavioral deficits exhibited in the cKO mice, we performed high-throughput RNA sequencing (RNA-seq) to identify genes with altered expressions when SENP2 is selectively removed in the forebrain excitatory neurons. RNAs were prepared from cerebral cortex tissues isolated from the brains of 6-week-old cKO and their littermate control mice. We sequenced RNA libraries from 3 biological replicates per genotype and evaluated the data by pearson correlation coefficient. Differential expression genes (DEGs) analysis revealed consistent changes between the two genotypes across all 3 replicates (Fig. 4a). We observed 863 up-regulated genes and 170 down-regulated genes in cerebral cortex of cKO mice using an adjusted *p* value < 0.05 and relative gene expression level > 2 fold change (Fig. 4b). A detailed comparative analysis of the gene expression profiles appears in Additional file 1: Table 1.

### GO and KEGG enrichment analysis for DEGs

**Fig. 5:**
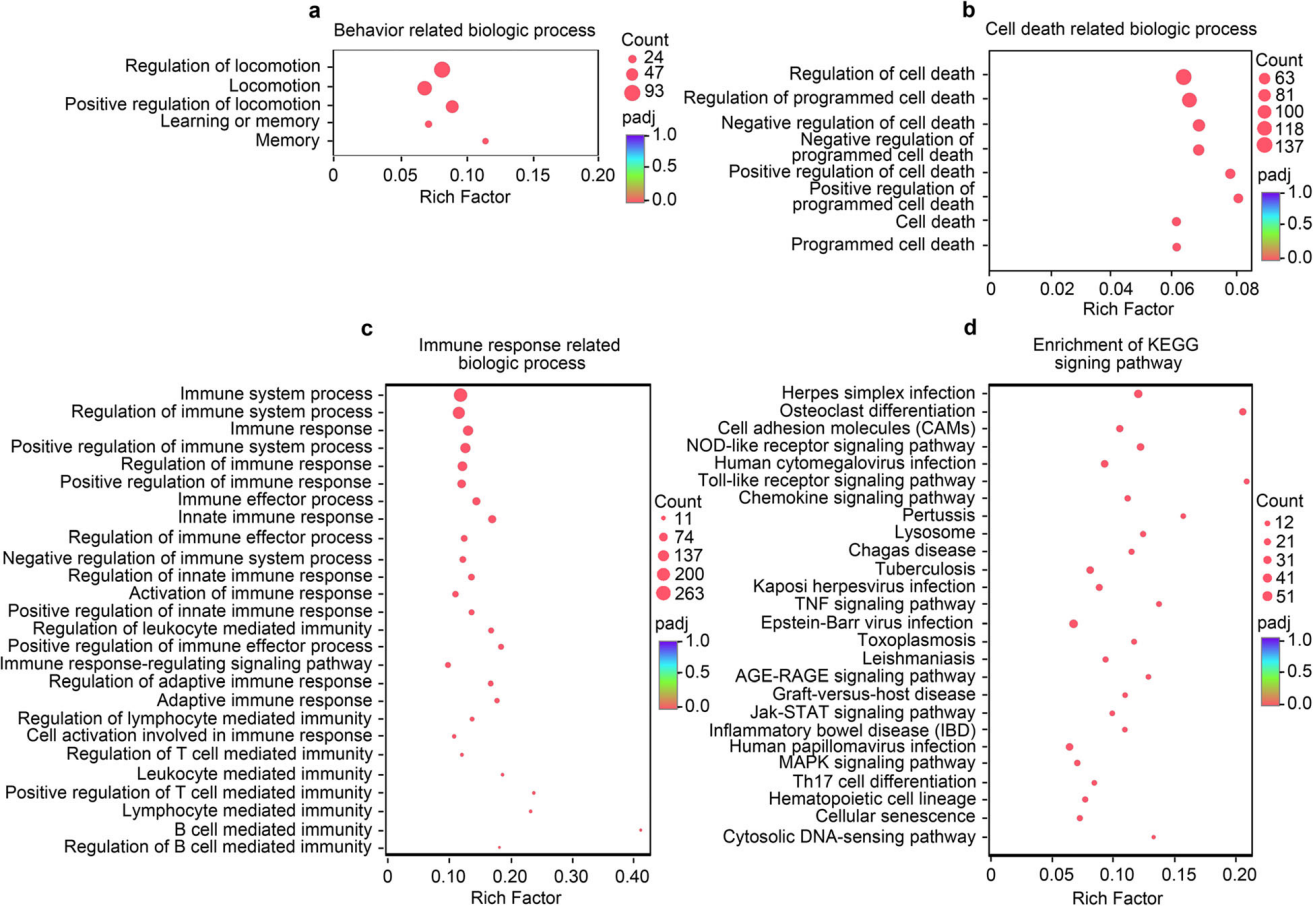
GO functional and KEGG pathway enrichment analysis of DEGs. **a**-**c** Separate GO enrichment analysis was carried out with “Goatools” using Fisher’s exact test and Benjamini-Hochberg was used to multiple testing corrections. The up-regulated and down-regulated genes were classified into different functional categories according to the GO term enrichment analysis for behavior related biological process, such as “Locomotion”, “Learning and memory” related biologic processes were enriched (**a**), cell death related biologic process, such as “Programmed cell death” related biologic processes were enrichment (**b**), Immune response related biologic process, such as “Immune response” related biologic processes were enrichment (**c**). **d** KEGG signing pathway enrichment analysis of differential expression genes. KEGG enrichment analysis was performed with “KOBAS 2.0” using Fisher’s exact test and Benjamini-Hochberg was used to multiple testing correction. The “Immune system”, “Cell growth and death” related pathway significantly enriched. Adjusted *p* value < 0.05 represents GO term or KEGG signing pathway significantly enriched. Rich factor means the ratio of enriched genes in background genes. The larger value of rich factor, the more enrichment of GO term or KEGG signing pathway. Count indicates the number of genes and “padj” means adjusted *p* value

Gene ontology (GO) analysis of the DEGs revealed SENP2 ablation altered the expression of genes involved in several biological processes, including locomotion, learning and memory (Fig. 5a). Additionally, GO analysis of DEGs revealed enrichment for numerous cellular and molecular functional categories, including those related to “Cell death” (Fig. 5b) and “Immune response” (Fig. 5c). A detailed GO enrichment analysis of the related GO terms is included in the Additional file 1: Table 2. Together, these results suggest that DEGs between cKO and littermate control mice are enriched for locomotion, learning and memory, cell death risk related genes. To further explore the molecular signaling pathway related to behavioral phenotypes, we introduce KEGG (Kyoto Encyclopedia of Genes and Genomes) enrichment analysis for DEGs (Fig. 5d). A detailed KEGG enrichment analysis of the KEGG signaling pathways appears in the Additional file 1: Table 3. KEGG enrichment analysis showed that “Immune system” related pathways were significantly enriched such as “Inflammatory bowel disease (IBD)”, “Th17 cell differentiation”, “Hematopoietic cell lineage”, “Cytosolic DNA-sensing pathway” and so on. These results were in accordance with the results of the GO enrichment analysis of “Immune response related biologic process” (Fig. 5c). On the other hand, we observed that “Cell growth and death” related pathways were also enriched such as “Cellular senescence” and “P53 signaling pathway”. These pathways may involve in modulating the programmed cell death of cKO mice [18–20]. Previous studies revealed that “MAPK signaling pathway” involved in regulating anxiety and depression-like behavior in mice [21, 22]. Our observation also showed that SENP2 ablation decrease anxiety-like behavior (Fig. 2a-o). Based upon the enriched KEGG pathways, previous studies and our findings, the “MAPK signaling pathway” could be one of a possible mechanism in modulating anxiety-like behavior in cKO mice.

### Multiple gene expression changes associated with anxiety-related behavior

**Fig. 6:**
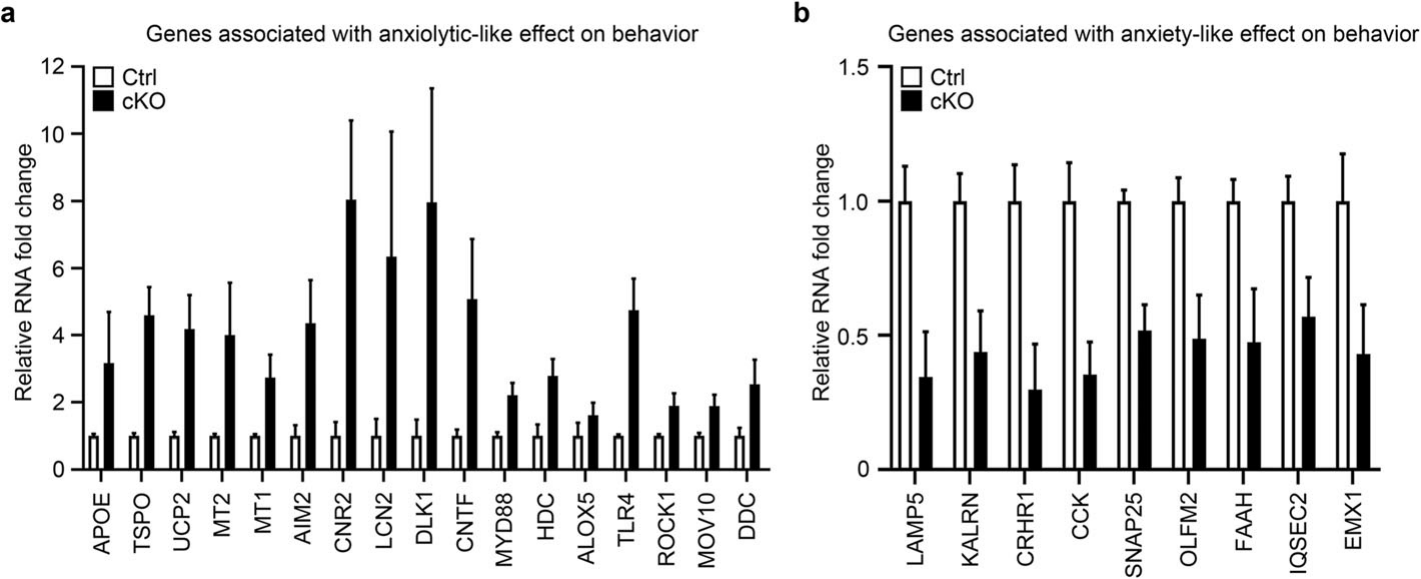
Multiple gene expression changes associated with anxiety-related behavior. 26 genes were associated with anxiety or anxiolytic-like effects on behavior based on 1033 DEGs. **a** 17 up-regulated genes associated with anxiolytic-like effects on behavior. **b** 9 down-regulated genes associated with anxiety-like effects on behavior. All data presented as mean ± S.E.M.

As the SENP2 cKO mice exhibit decreased anxiety-like behavior (Fig. 2), we attempted to screen out the genes associated with anxiety-like behavior from DEGs. However, to the best of our knowledge, there are no available databases that record anxiety disorder related genes. Thus, we decided to retrieve the 1033 DEGs using the PubMed database. By searching for the literature, we identified that 17 genes associated with anxiolytic-like effect on behavior are up-regulated (Fig. 6a), and 9 genes associated with anxiety-like effect on behavior are down-regulated (Fig. 6b). In addition, the 26 genes of anxiety-like or anxiolytic-like effect on behavior have been further confirmed by transgenic mice. Taken together, these genes may have an important role in modulating the level of anxiety. The 17 genes associated with anxiolytic-like effect on behavior, including that APOE [23], TSPO [24], UCP2 [25], MT1 [26], MT2 [27], AIM2 [28], CNR2 [29], LCN2 [30], DLK1 [31], CNTF [32], MYD88 [33], HDC [34], ALOX5 [35], TLR4 [36], ROCK1 [37], MOV10 [38], DDC [39]. The 9 genes associated with anxiety-like effect on behavior, including that LAMP5 [40], KALRN [41], CRHR1 [42], CCK [43], SNAP25 [44], OLFM2 [45], FAAH [46], IQSEC2 [47], EMX1 [48]. These data suggest that either the anxiolytic-like effects gene up-regulated or anxiety-like effects gene down-regulated could be a reduced level of anxiety. However, to elucidate the detailed mechanism of decreased anxiety-like behavior, further studies are needed to screen and determine from the 26 candidates and confirm its role in regulating the level of anxiety in cKO mice.

## Discussion

Animal models are extremely useful in establishing causality between genetic mutations, synaptic changes, circuit dysfunctions and abnormal behaviors, thereby aiding us in understanding the pathogenesis of neurological and neuropsychiatric diseases [49, 50]. SENP2 is a member of the sentrin/SUMO-specific proteases (SENPs) family and implicated in embryonic development [6, 10], fatty acid metabolism [51], atherosclerosis [52], and epilepsy [8]. However, the function of SENP2 in CNS and its potential contribution to the neuropathology remains unclear. In this study, we generated conditional knockout of SENP2 in excitatory neurons in the postnatal forebrain and determined that these cKO mice display comprehensive behavioral phenotypes including hyperactivity, reduced anxiety-like behavior, impaired learning and memory (Figs. 2 and 3). Consistently, RNA-seq results showed that the loss of SENP2 is associated with moderate changes in gene transcripts related to “locomotion”, “learning and memory” (Fig. 5a) and multiple gene expression changes associated with anxiety-related behavior (Fig. 6). In addition, GO enrichment analysis identified changes in genes related to “Cell death” and “Immune response” (Fig. 5b-c). Taken together, our results demonstrate that SENP2 plays important functional roles in the forebrain.

SUMOylation is a dynamic and reversible posttranslational protein modification that regulates the functions of target proteins [53]. In the CNS, neuron-specific SUMO1–3 knockdown mice show less exploration of center area in open field test [12]. Additionally, previous studies showed that hippocampus-dependent learning and memory is impaired by overexpression of a dominant negative Ubc9 peptide in the hippocampal CA1 area [13]. These results suggested that the balance of SUMOylation/deSUMOylation is critical for forebrain mediated function, including hippocampus-dependent learning and memory, and anxiety-like behaviors. SENPs catalyze the removal of SUMO from SUMO-conjugated proteins, thus playing a critical role in regulating the SUMOylation level of targets [2, 3]. Previous study revealed that neuron-specific SENP2 knockout mice display hyperactivity and sudden death [8]. Consistently, we report here that the forebrain excitatory neuron-specific SENP2 knockout mice displayed hyper-locomotor activity in the open field test. Moreover, using several different behavioral assays, we identified a reduced anxiety phenotype in the SENP2 cKO mice (Fig. 2j-o).

Previous study has reported that death of matured neurons in the forebrain increases the level of anxiety [54]. To further explore the molecular mechanism related to behavioral phenotypes of cKO mice, we conducted RNA-seq on the cortex of cKO animals and observed that numerous DEGs related to programmed cell death biologic processes were enriched (Fig. 5b), suggesting that ablation of SENP2 may lead to neuronal death. However, we don’t yet have any direct evidence showing that conditional knockout of SENP2 led to programmed cell death in the forebrain excitatory neurons. Further study is certainly needed to investigate the potential linkage between neuronal death and anxiolytic-related behaviors in the SENP2 cKO mice. On the other hand, we identified 26 genes from 1033 DEGs that are implicated in anxiety-related behaviors. These include 17 up-regulated genes known to have anxiolytic-like effects and 9 down-regulated genes have anxiety-like effects (Fig. 6a-b). This finding thus should shed some light on the potential molecular mechanism for the anxiolytic behaviors exhibited by the SENP2 cKO mice.

The SENP2 cKO mice also exhibited deficits in associative learning and spatial working memory functions, which were assessed in both contextual fear conditioning test and Y-maze spontaneous alternation task respectively (Fig. 3a-b). Consistent with the observed cognitive impairments, the RNA-seq studies we conducted on the cortex of cKO animals detected 20 DEGs that are specifically related to learning and memory-related tasks (Fig. 5a). These findings suggest that SENP2-dependent protein modifications are important for learning and memory, and dysfunction of SENP2 contributes to impaired cognitive functions in mice. Moreover, GO analysis of the DEGs in the SENP2 cKO mice revealed enrichment of genes for several cellular and molecular functional categories, including those related to “Cell death” and “Immune response” (Fig. 5b-c). Given that SENP2 has been implicated in neural disorders such as epilepsy and neurodegeneration [7, 8], future studies should be directed to examine the role of these SENP2-regulated molecular pathways in the observed behaviors.

As the subcellular localization of SENP2 is predominantly nuclear, we performed the differential analysis of gene and transcript expression using high throughput RNA-seq. These analyses suggest that SENP2 knockout results in changes of gene transcription that are associated with various behaviors including locomotion, learning and memory, anxiety. One interpretation of these results is that SENP2 dependent post translational modification of proteins, such as transcription factors and co-factors, may lead to alterations in gene expression. However, it is unclear whether these changes in gene transcription are directly caused by the loss-of-function of SENP2. On the other hand, it is very likely that SENP2 can directly regulate neuronal functions by altering the status of SUMOylated proteins. We hope that, in future studies, different proteomics approaches will help further gain insight into the role of SENP2 plays in the CNS.

In summary, we generated a line of cKO mice to investigate the functions of SENP2 in the CNS. Our results demonstrate that dysfunction of SENP2 in forebrain excitatory neurons leads to behavioral changes associated with emotional and cognitive functions.

## Materials and methods

### Experimental animals

All animal procedures were carried out in accordance with the guidelines for care and use of laboratory animals of Shanghai Jiao Tong University School of Medicine and approved by the Institutional Animal Care and Use Committee (IACUC). All mice used in this study were C57BL/6 background. SENP2^fl/fl^ mice [8] were crossed with CaMKIIα-Cre mice [11] to generate SENP2 conditional knockout mice (cKO). PCR primers used for genotyping are the following: SENP2 loxP Forward: 5′-CTTCTGCTTCTCTTAGTGCT-3′ and SENP2 loxP Reverse: 5′-CTTCTGCTTCTCTTAGTGCT-3′, with the expected product sizes of 183 and 149 bps for SENP2^fl/fl^ and WT mice respectively. The presence of CaMKIIα-Cre was identified by PCR using the following primers: Cre Forward: 5′-CGCTGGGCCTTGGGACTTCAC-3′ and Cre Reverse: 5′-CAGCATTGCTGTCACTTGGTC-3′, with the expected PCR product size of 403 bps.

### Immunofluorescence staining

Immunofluorescence staining was performed as described previously [55], with minor modifications. Mice were perfused intracardially with 4% paraformaldehyde. After an overnight postfixation in the same fixative at 4 °C, brain tissues were embedded in 2% agar and cut into 50 μm sections with a vibratome (Leica, VT1200S). Brain sections were blocked with 0.3% Triton X-100 and goat serum in PBS for 1 h at room temperature and then incubated with SENP2 (1:100, Abcam, ab58418) and CaMKIIα (1:400, ThermoFisher Scientific, MA1–048) primary antibody overnight at 4 °C. Next, brain sections were incubated with Alexa Fluor546-conjugated goat anti-rabbit (Invitrogen, 1:500, A-11035) or Alexa Fluor488-conjugated goat anti-mouse secondary antibodies (Invitrogen, 1:500, A-11029) for 1 h at room temperature and mounted on glass slides using a small brush. The fluorescence images were acquired using the confocal microscope (Leica, TCS SP8).

### Western blot

Western bolt was performed as previously described with minor modifications [4]. Brain tissues were collected on ice and homogenized with lysis buffer I (50 mM Tris-HCl, pH 6.8, 2% SDS, 40 mM DTT, and 5% glycerol). After denaturation for 15 min at 95 °C, the samples were diluted 10-fold with lysis buffer II (50 mM Tris-HCl, pH 7.4, 150 mM NaCl and 1% NP-40) and ultra-sonicated for 5 s, followed by centrifugation for 10 min at 13,000×g (4 °C). The supernatant was transferred to a new tube and boiled with loading buffer for 15 min. The proteins were separated by SDS-PAGE, transferred to polyvinylidene fluoride (PVDF) membranes, blocked with 5% non-fat milk, and immunoblotted with SENP2 (1:1000, Santa Cruz, sc-376,731) and β-actin (1:10000, Santa Cruz, sc-130,065) antibodies.

### Real-time quantitative polymerase chain reaction (qPCR)

qPCR was performed as previously described with minor modifications [5]. Brain tissues (cortex, hippocampus and cerebellum) were isolated on ice from cKO and littermate control mice. The total RNAs were extracted from these brain tissues using TRIzol Reagent (Tiangen, Beijing, China). After removing genomic DNAs, the total RNAs were reverse transcribed to complementary DNA (cDNA) using the PrimeScript RT reagent Kit (Takara, Dalian, China) according to the manufacturer’s protocol. The cDNAs were then used as templates for the qPCR reactions, which were performed in a 10 μl volume with Power SYBR Green PCR Master Mix (CWBIO, Beijing, China) and 0.2 μM primers using the LightCycler 480 real-time PCR system (Roche, CA, USA). The qPCR primers used for cKO and littermate control mice were as follows: SENP2 forward: 5′-TTCTCGGCACCATTCTTCGCTTGT-3′, SENP2 reverse: 5′-TGCTGCAGGATCCAGAACTCATCA-3′. GAPDH forward: 5′-CATGGCCTTCCGTGTTCC-3′ and GAPDH reverse: 5′-GCCTGCTTCACCACCTTCTT-3′.

### RNA sequencing (RNA-seq)

RNA extraction: Three pairs of cortical tissue were isolated on ice from 6-week-old cKO and littermate control mice and quickly frozen in liquid nitrogen. The samples were delivered to the company (Majorbio Bio-pharm Technology, Shanghai, Chain) to prepare RNA samples and conduct high-throughput RNA-seq. The RNA quality was determined by 2100 Bioanalyser (Agilent Technologies, Tokyo, Japan) and quantified using the ND-2000 (NanoDrop Technologies, DE, USA). The RNA integrity was also confirmed with agarose gel electrophoresis.

Library construction and sequencing: 5 μg high-quality RNA samples were used to construct sequencing library. Briefly, messenger RNA was isolated from total RNA samples and then fragmented by fragmentation buffer firstly. Secondly, double-stranded cDNA was synthesized using a SuperScript double-stranded cDNA synthesis kit (Invitrogen, CA, USA) with random primers (Illumina, CA, USA). Thirdly, the synthesized cDNA was subjected to end-repair, phosphorylation and ‘A’ base addition according to Illumina’s library construction protocol. Then, 200–300 bp cDNA target fragments were isolated using 2% agarose followed by PCR amplified, the isolated cDNA fragments were selected to construct sequencing library. Libraries quantified by TBS380, and sequenced with the Illumina HiSeq 4000 (Illumina, CA, USA).

Read mapping: The raw paired end reads were trimmed by “SeqPrep” software and quality controlled by “Sickle software. Then clean reads were separately aligned to reference genome with orientation mode using “TopHat” software. The criteria for mapping were that sequencing reads should be uniquely matched to the genome with less than 3 mismatches, without insertions or deletions. Then, the gene regions were expanded following depths of sites and the operon was obtained. In addition, the whole genome was segmented into multiple 15 kb fragments that share the same 5 kb fragments. If more than 2 consecutive fragments were without overlapped region and at least 2 reads mapped per fragment in the same orientation, we consider it was new transcribed region of gene [56].

Data analysis: To identify differentially expressed genes (DEGs) between cortical tissues of cKO and littermate control mice, each transcript expression level was calculated according to the fragments per kilobase of transcript per million mapped reads (FPKM) method. The gene abundances were quantified using the RSEM software, while the EdgeR software was used for differential expression analysis and quantified transcript read counts. In addition, the differential expression analysis was carried out on an online platform (www.majorbio.com) followed by multiple check calibration (BH). A differentially expressed gene is identified as relative read counts > 2 fold change and adjusted *p* value < 0.05. Similarly, Go enrichment analysis was conducted using the “Goatools” software followed by Fisher’s exact test. KEGG enrichment analysis was using the “KOBAS 2.0” software followed by Fisher’s exact test. If the adjusted *p* value < 0.05, we consider the GO terms or KEGG signing pathways were significantly enriched.

### Experimental design for behavioral tests

Behavioral tests were performed on male mice of 8 to 12-weeks of age. Mice were housed in a room with 12 h light/dark circadian rhythm, suitable temperature (22–28 °C), adequate water and food. The behavioral tests were performed in the light-on phase of the cycle (10:00 a.m.-18:00 p.m.) except for otherwise noted. The experimenters were blinded to the genotype of each mouse during all tests and data analyses. The tests were performed in the following sequence: open field, elevated plus maze, novelty suppressed feeding, nest building, Y maze, and contextual fear condition. The startle response/prepulse inhibition test was performed using another set of male mice without any stressors. Tests were repeated using at least two different cohorts of mice. These tests were performed at intervals of 2–4 days.

### Open field test

The open field test was performed as previously described with minor modifications [57]. Mice were habituated in the testing room for 60 min and then introduced to the open field apparatus (40 × 40 × 30 cm) (MED Associates). The test mice were allowed to freely explore for 30 min in apparatus without interference. The distance traveled, number of entries in center area (20 × 20 cm), time spent in center area and distance traveled in center area were automated recorded by monitor system and software (EthoVision XT 12).

### Elevated plus maze

Elevated plus maze test was performed as previously described [58]. Mice were habituated in the testing room for 60 min and placed in the intersection of open and closed arms with the mouse head toward the open arm. The test mice were allowed to freely explore for 5 min in the apparatus (MED Associates) without interference. Monitor system and software (EthoVision XT 12) automated recorded the time that mice spent in open and closed arms as well as the number of entries in open and closed arms respectively.

### Novelty suppressed feeding

Novelty suppressed feeding test was performed as previously described [59] with minor modifications. Briefly, mice were fasted for 24 h in the home cage before testing and then placed in a new feeding box in which food was fixed on one piece of round filter paper (10 cm in diameter) at the center of the plastic chamber (30 × 40 cm). The mice were allowed to freely explore and eat the food for 5 min. A monitor system (EthoVision XT 12) recorded the process of food eating including the latency to feeding.

### Y maze

The Y maze has three identical opaque arms (40 cm-long, 10 cm-wide, and 15 cm-high). We conducted the Y maze test as previously described [60]. Briefly, mice were habituated the testing room for 60 min and placed in the distal end of one arm. The mice were allowed to freely explore for 5 min without any interference. The monitor system (EthoVision XT 12) recorded the locus of mice movement, while the sequence of entries was manually recorded and analyzed by the experimenter. The alternation ratio was calculated as follows:

$$ \mathrm{Alternation}\kern0.3em \left(\%\right)=\mathrm{Alternated}\kern0.3em \mathrm{number}\mathrm{s}/\left(\mathrm{Total}\kern0.3em \mathrm{number}\kern0.3em \mathrm{of}\kern0.3em \mathrm{entries}\kern0.3em \mathrm{in}- 2\right)\kern0.3em \mathrm{x}\kern0.3em 100\%. $$

### Contextual fear condition

Contextual fear condition was conducted as previously described with minor modifications [61]. Briefly, mice were habituated in the testing room for 60 min and then placed in a test chamber with a black and white plaid sticker on all sides for 10 min to habituate the apparatus (Ugo Basile) that constantly presents 100 lx bright light for 2 consecutive days. In the training session, mice were placed in the test chamber with a metal grid floor and received footshock (0.5 mA, 2 s) for 5 trials with 120 s interval. After the footshock, mice remained in the conditioning chamber for another 30 s and then were placed back to their home cage for 24 h. In the retrieval session, mice were placed into the same contextual chamber for 10 min test without footshock. The freezing values were recorded every 2-min block by the monitor system and software (ANY-maze).

### Nest building

Nest building test was performed as previously described with minor modifications [62]. Briefly, mice were individually housed for 12 h (20:00 p.m.-8:00 a.m.) while providing a piece of cleansing paper into the cage. The next day, the nesting score was assessed based on the integrity of the paper by an experimenter blinded to mouse genotypes [63]. Nests were given a score of 0–5 according to the following criteria: 1 = more than 90% of paper was intact; 2 = 50–90% of paper remained intact; 3 = more than 50% of paper was torn, but no identifiable nest site; 4 = more than 90% of paper was torn and a flat nest was built; 5 = more than 90% of paper was torn and the paper was transformed into a tridimensional nest.

### Prepulse inhibition (PPI)

Prepulse inhibition test was carried out as previously described [64] with minor modifications. Mice were habituated to the testing room for 60 min, and placed into the test chamber for 5 min to acclimate the apparatus (MED Associates) that present the constant background white noise of 65 dB for 2 consecutive days. In stage I of the PPI session, we replaced the mice into the chamber for a 5 min acclimation. In stage II of PPI session, we presented 10 trials of high acoustic stimulus (120 dB) with an interval of 20 ms to make the mice accommodate the high acoustic stimulus. In stage III of PPI session, we random presented seven types of acoustic stimulus including: 1) the high acoustic stimulus (120 dB) only or 2) the low acoustic stimulus only (70 dB, 74 dB and 78 dB) or 3) the high acoustic stimulus paired with a low acoustic stimulus (70 dB paired with 120 dB or 74 dB paired with 120 dB or 78 dB paired with 120 dB) with a random interval of 30–100 ms. In our protocol, six blocks containing seven acoustic stimulus types were presented in a pseudorandom order and each acoustic stimulus type was presented once within a block. The acoustic reflex was measured by SR-Lab system (San Diego Instruments) and counted using the largest peaks of amplitude that the signal recorded in a 300 ms window after presented the acoustic stimulus. We respectively counted the startle amplitude of high acoustic stimulus (120 dB only) and the paired acoustic stimulus (70 dB paired with 120 dB or 74 dB paired with 120 dB or 78 dB paired with 120 dB). The startle amplitude of 120 dB stimulus represented the acoustic reflex of mice, and was used as the baseline value of PPI. The PPI was calculated as follows:

$$ \mathrm{PPI}\kern0.3em \left(\%\right)=\left(\mathrm{Startle}\kern0.3em \mathrm{amplitude}\kern0.3em \mathrm{of}\kern0.3em 120\kern0.3em \mathrm{dB}\kern0.3em \mathrm{acoustic}\kern0.3em \mathrm{stimulus}-\mathrm{Startle}\kern0.3em \mathrm{amplitude}\kern0.3em \mathrm{of}\kern0.3em \mathrm{paired}\kern0.3em \mathrm{acoustic}\kern0.3em \mathrm{stimulus}\right)/\left(\mathrm{Startle}\kern0.3em \mathrm{amplitude}\kern0.3em \mathrm{of}\kern0.3em 120\kern0.3em \mathrm{dB}\kern0.3em \mathrm{acoustic}\kern0.3em \mathrm{stimulus}\right)\kern0.3em \mathrm{x}\kern0.3em 100\%. $$

### Statistical analysis

All data are presented as mean ± S.E.M and analyzed by GraphPad Prism8 software. Two-group comparison was processed with two-tailed, unpaired Student’s *t* test when the variance is equal (Fig. 2e, g, i, l, o, Fig. 3a, c) or Welch’s *t*-test when the variance is unequal (Fig. 2c, k, m, n). Multiple group comparison was performed with two-way ANOVA analysis followed by Bonferroni’s post-hoc to determine significance (Fig. 1d-e, Fig. 2b, d, f, h, Fig. 3b, d). *p < 0.05, **p < 0.01, ***p < 0.001 compared with littermate controls.

### Data availability statement

The data that support the findings of this study are available from the corresponding author upon reasonable request.

## Supplementary information

**Additional file 1: Table S1.** List of DEGs (Differentially Expressed Genes) from RNA-Seq analysis results. **Table S2.** List of GO term from RNA-Seq analysis results. **Table S3.** List of KEGG signing pathway from RNA-Seq analysis results.
